# Influence of Rapid Heat Treatment on the Shrinkage and Strength of High-Performance Concrete

**DOI:** 10.3390/ma14154102

**Published:** 2021-07-23

**Authors:** Jan Stindt, Patrick Forman, Peter Mark

**Affiliations:** Institute of Concrete Structures, Ruhr University Bochum, 44801 Bochum, Germany; patrick.forman@rub.de (P.F.); peter.mark@rub.de (P.M.)

**Keywords:** heat treatment, high-performance concrete, shrinkage, compressive strength, rapid flow production

## Abstract

Resource-efficient precast concrete elements can be produced using high-performance concrete (HPC). A heat treatment accelerates hardening and thus enables early stripping. To minimise damages to the concrete structure, treatment time and temperature are regulated. This leads to temperature treatment times of more than 24 h, what seems too long for quick serial production (flow production) of HPC. To overcome this shortcoming and to accelerate production speed, the heat treatment is started here immediately after concreting. This in turn influences the shrinkage behaviour and the concrete strength. Therefore, shrinkage is investigated on prisms made from HPC with and without steel fibres, as well as on short beams with reinforcement ratios of 1.8% and 3.1%. Furthermore, the flexural and compressive strengths of the prisms are measured directly after heating and later on after 28 d. The specimens are heat-treated between 1 and 24 h at 80 °C and a relative humidity of 60%. Specimens without heating serve for reference. The results show that the shrinkage strain is pronouncedly reduced with increasing temperature duration and rebar ratio. Moreover, the compressive and flexural strength decrease with decreasing temperature duration, whereby the loss of strength can be compensated by adding steel fibres.

## 1. Introduction

Constructions with precast concrete elements like trusses, walls, tunnel lining segments [1], or beams (modules) made of high-performance concrete (HPC) [2,3] with a compressive strength between 55 and 100 MPa have been found to be advantageous compared to conventional cast-in place members. Elements are prefabricated independently of weather conditions and in a time-saving manner. Due to the high load-bearing capacity of HPC, filigree and slender elements arise. Their durability increases due to the dense pore structure of HPC [4]. The precast elements are assembled on-site via dry joints, bolted connections or socket joints, for example, so that construction times are shortened, and costs are reduced [5]. Since there are no tolerance compensating joints due to a direct force transmission, fast installation depends essentially on the shape stability of the concrete elements [6].

The main, load-independent deformations of concrete result from shrinkage, which is divided into drying, chemical, autogenous, and plastic shrinkage. Plastic shrinkage occurs in fresh concrete during hydration due to evaporation [7]. The capillary forces that arise during evaporation cause a volume change [8]. In drying shrinkage, the volume change is caused by the evaporation of the free pore water from the concrete matrix due to the low external ambient humidity [9].

In autogenous shrinkage, the volume change is caused by the loss of free water in the concrete matrix due to hydration [7]. The hydration products require a lower volume fraction (chemical shrinkage) [10]. The combination of chemical and autogenous shrinkage (basic shrinkage) governs the overall shrinkage extent of HPC due to the fine pore structure and the low water–binder (w/b) ratios.

Next to the w/b ratios [11,12], basic shrinkage also depends on the aggregates [13], the supplementary cementitious material, and the steel fibres. For example, blast furnace slag exhibits a finer pore structure than ordinary Portland cement and increases the shrinkage strain [13,14]. Steel fibres reduce the basic shrinkage through their volume fraction and shape [15]. The higher Young’s modulus supports the crystal grating of the clinker phases [13]. Furthermore, calcium hydroxide crystals grow on the surface of the fibres, what loosens the structure of the concrete matrix [13]. Moreover, the contact zone between concrete and fibres shows lower amounts of clinker, which increases the porosity [16]. This facilitates moisture penetration and affects drying shrinkage. Then increased strains due to drying shrinkage are observed, since the steel fibres, among other aspects, connect the pores in the concrete and lead to increased water diffusion [17].

Heat treatment anticipates significant amounts of the basic shrinkage, since the cement largely hydrates already during the treatment [18]. The specific influences on shrinkage depend on many factors such as the concrete’s composition [12] and can vary greatly [19]. In general, shrinkage is assumed to be almost completed after heat treatment [20].

In addition, heat treatment is used to accelerate the development of early strength and thus shortens the curing time and reduces the production cycle of precast concrete. Compared to concrete without heat treatment, the long-term strength is reduced by increased temperatures. This results from micro-damages due to the different thermal expansion coefficients of the hardened cement paste, the aggregates, and air pores [21]. It increases with the duration of heating [22]. Structural damage can occur due to shrinkage cracks [23] and internal stresses as a result of heat treatment [24]. Moreover, the durability of the concrete can be impaired by the formation of secondary ettringite [25]. To limit that disadvantage, heat treatment follows a strict schedule. It consists of a pre-storage and a heating phase, followed by a dwell and a cooling phase [25]. Ettringite decomposition happens at temperatures around 70 °C [26], so that the maximum temperature of heating according to Deutscher Ausschuss für Stahlbeton (DAfStb) guideline and American Concrete Institute (ACI) guideline [25,27] is limited between 60 to 80 °C. Whereby 80 °C is only used for permanently dry precast concrete elements. After concreting, a pre-storage time of 3 to 5 h is planned to ensure sufficient tensile strength against microcracks. For the same reason, the heating and cooling rates are limited between 20 to 33 K/h [25,27]. Despite the measures described above, the compressive strength is reduced up to 18% and the flexural strength up to 30% as a result of heat treatment [28]. Thus, the overall duration of heat treatment usually takes between 17 and 33 h.

For HPC, the requirements of heat treatment are similar to those of ordinary concrete. According to [29], heating temperatures between 70 °C and 90 °C are suggested for ultra-high-performance concrete (UHPC) with durations of 48 h and an additional one-day pre-storage period. In [30], UHPC is heat-treated between 90 and 200 °C for 78 h and at least one-day pre-storage. With the same pre-storage time, dwell times between 1 and 4 d at 60 °C and between 12 h and 48 h at 80 °C are proposed in [22]. 

An increased compressive strength due to heat treatment is achieved in [22,29,30] for UHPC with silica dust. Addition of silica enables further pozzolanic reactions that lead to formation of additional calcium silicate hydrates (C–S–H). Thus, the silica dust densifies the pore structure and reseals occurring microcracks [4]. The compressive strength can be further increased by activating the non-hydrated binder in post-treatment, e.g., by water storage [20]. Alternatively, steel fibres counteract strength reductions by absorbing tensile stresses and preventing the growth of microcracks [4,15,31]. The benefit of fibres on the compressive and flexural strength mainly depends on the shape, the dosage, and their orientation [32,33,34]. Microfibres [35], in particular, can be used with higher volume fractions and distribute more evenly than macrofibres with hooked ends in the concrete matrix [15,17]. 

In this article, the shrinkage behaviour and strength development of rapidly heat-treated HPC with and without steel fibres [36] and with reinforcing bars is experimentally investigated. Both the heating rate and pre-storage time are unlimited. The paper starts from the experimental campaign of small-scale samples in which heating time, fibre additions, and rebar amounts are varied. Shrinkage is isolated from thermal strains. Finally, strength values are provided and discussed relative to those of reference samples without heat treatment.

## 2. Materials and Methods

The investigations aim to empirically determine the shrinkage strain *ε*_cs_ and the compressive and flexural strengths regarding the temperature duration *T*. The measurements are performed on prisms (*L* × *W* × *H* = 16 × 4 × 4 [cm]) without steel fibres and a steel fibre amount of *V* = 150 kg/m³ (1.9 Vol.-%). For comparison, beams with a fibre amount of 150 kg/m³ and reinforcement ratios of 1.8% and 3.1% are investigated, too. They serve to check the influence of rebars on the shrinkage strain. The reinforcement ratios reflect diameters of 6 mm and 8 mm, respectively. To ensure sufficient anchorage length, the length of the associated beams *L* has been increased to 40 cm. An HPC based on the binder Nanodur^®^ Compound 5941 [37] is used for all specimens. The mixture is listed inTable 1. 

Directly after concreting, the prisms and the beams are subjected to heat treatment at 80 °C and 60% relative humidity. Short temperature durations of *T* = [1, 2, 4, 6] h, but also long durations of *T* = 24 h are investigated. Thereby, the temperature duration *T* includes the heating phase and the dwell time. Specimens without heat treatment (*T* = 0 h) serve for reference. After heat treatment, the test specimens—six per duration and type—are stripped and stored at 20 °C and 65% relative humidity for up to 90 days. 

For each *T*, six beams and prisms are made independently of the rebar ratios *r* and steel fibre amount *V*. The series comprises 72 prisms and beams in total including the references. Table 2 lists all and provides the number of tested specimens in each case at the given concrete age after heating depending on the total number of samples. In parallel, 12 prisms with and without steel fibres for *T* = 2–24 h are made to determine the short-term mechanical properties. In total, this sums to 48 prisms without reinforcement, 120 with microfibres (incl. beams) and 72 beams with rebar and fibres. 

### 2.1. Temperature Measurement

**Figure 1:**
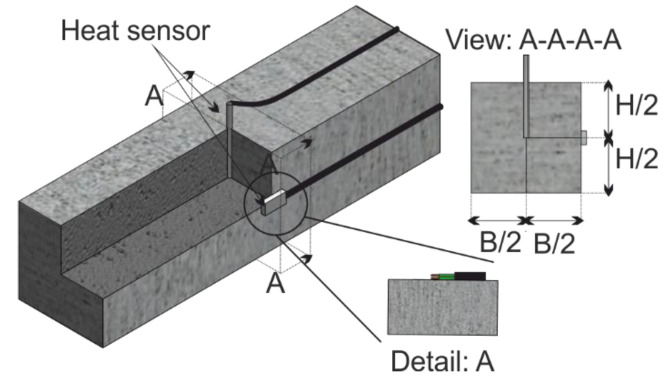
Experimental set-up for the temperature measurement.

Due to heating, temperature strains occur that superimpose with the shrinkage strains. Therefore, the temperatures at the core and at the surface of selected prisms are measured. Figure 1 shows the test set-up for the temperature measurement. A heat sensor is embedded at the core of the prism for each temperature duration *T* and fibre amount *V*, to measure the core temperature *ϑ*_m_ (section: A-A-A-A). After stripping, another sensor is glued on the face to measure the surface temperature *ϑ*_w_ during cooling (Figure 1, Detail: A).

### 2.2. Shrinkage Measurement

**Figure 2:**
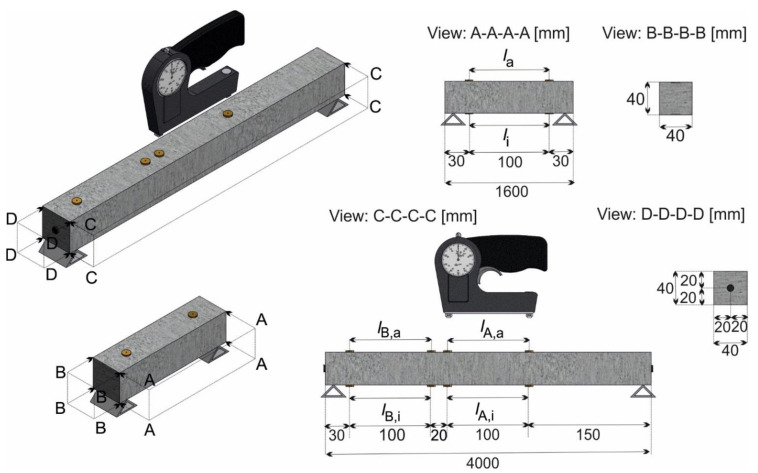
Experimental set-up for the shrinkage measurement.

Shrinkage deformation is determined daily up to day 28 and then weekly up to day 90. A stress-measuring extensometer type “Pfender” (TESTING Bluhm & Feuerherdt GmbH, Berlin, Germany) with an accuracy of up to 1 μm is used. Figure 2 shows the experimental set-up of the measurement for the samples. For both, markers with an initial length of 100 mm were glued on the specimens directly after heat treatment. For the prisms, two measuring sections are provided on the upper *l*_a_ and lower side *l*_i_ (section: A-A-A-A). To measure shrinkage strains representative for the entire length of the beams, the central measuring sections *l*_A,a_ and *l*_A,i_ are supplemented by two more sections with the lengths *l*_B,a_ and *l*_B,i_ (cf. section: C-C-C-C). The alteration of length in the individual sections is evaluated according to [38]. The mean over all sections of a sample yields the length *R*_t_ at concrete age *t*. The first is the zero measurement *R*_0_ at time *t*_0_^*^. The shrinkage strain ε_cs_ at time *t* is calculated to:

(1) $$\varepsilon_{\mathrm{cs},t}=\frac{∆l}{l}=\frac{R_{t}-R_{0}}{l}.$$

### 2.3. Strength Measurement

Compressive and flexural strength is derived from prism tests. After 28 days, flexural *f*_ct,fl,28d_ [39] and compressive strength *f*_cm,28d_ [40] are determined from three prisms for each *T* and *V*. For the short-term strength, an additional series of three prisms with and without fibres is used (cf. Table 2). The flexural *f*_ct,fl,0d_ and compressive strength *f*_cm,0d_ are measured directly after heat treatment.

## 3. Results

### 3.1. Results of the Temperature Measurement

**Figure 3:**
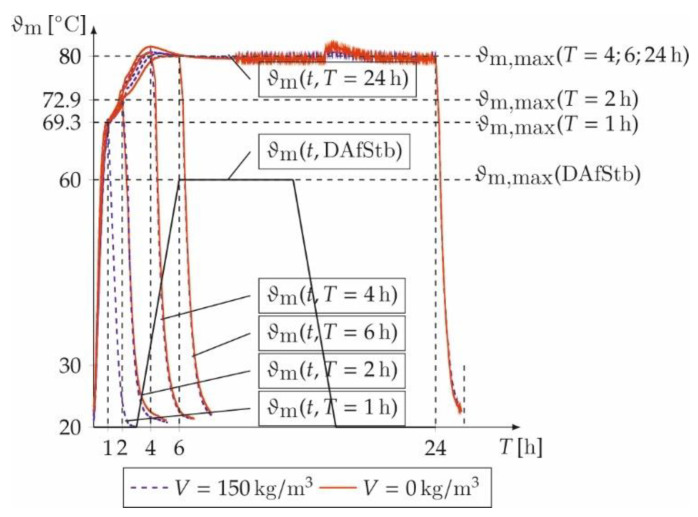
Core temperature ϑ_m_ of the prisms with and without fibres as well as the annealing regime according to [25].

The core temperature *ϑ*_m_ of the prisms during heat treatment is recorded and evaluated. Figure 3 shows a nonlinear increase and decrease of *ϑ*_m_ as well as a constant plateau during the dwell time. For comparison, the temperature curves of the prisms with (*V* = 150 kg/m³) and without (*V* = 0 kg/m³) fibres are shown for annealing times of *T* = 1, 2, 4, 6, and 24 h along with the achieved maximum core temperatures *ϑ*_m,max_ and the shortest heat treatment times according to DAfStb guideline [25]. The prism without steel fibres and a treatment time of *T* = 1 h broke during the measurement. Thus, Figure 3 just shows the prisms with steel fibres for *T* = 1 h. The essential differences are the elimination of the pre-storage times and the faster heating rate *R*_A_. The investigated heat treatment including the cooling phase is accelerated up to 86.8% (*T* = 1 h) and 67.2% (*T* = 6 h) in comparison.

The average heating rate *R*_A_ is calculated to

(2) $$R_{A}=\frac{\vartheta_{m,\max}-\vartheta_{0}}{t_{A}}.$$

Here, *ϑ*_m,max_ represents the maximum core temperature, *ϑ*_0_ the initial temperature of 20 to 23 °C and *t*_A_ the time until the maximum core temperature is reached. Based on the measured core temperatures of the prisms without fibres, heating rates of *R*_A_ = [45.9, 24.9, 15.9, 16.4, 16.3] K/h and *R*_A_ = [47.1, 25.5, 15.6, 16.5, 16.6] K/h with fibres for *T* = [1, 2, 4, 6, 24] h are achieved. 

It must be noted that for a temperature duration of *T* = 1 h (*ϑ*_m,max_ = 69.28 °C) and *T* = 2 h (*ϑ*_m,max_ = 72.92 °C) the intended temperature of 80 °C in the core was not reached. *ϑ*_m_ rises linearly for all samples to approx. 70 °C and then nonlinearly to *ϑ*_m,max_. The linear increase results from the free water in the concrete matrix that significantly influences the moisture-dependent thermal properties such as thermal conductivity and heat capacity [41]. The nonlinear increase results from hydration and binding of free water and reduces the heat capacity up to 15% and the thermal conductivity in the course of hydration by 10% [42]. The temperature profile of the prisms with and without fibres differs marginally. This is attributed to the low impact of steel fibres on the thermal properties of concrete [43,44]. Hence, to limit the heating rates, temperature durations of *T* > 2 h are recommended for the heat treatment presented here.

### 3.2. Shrinkage

**Figure 4:**
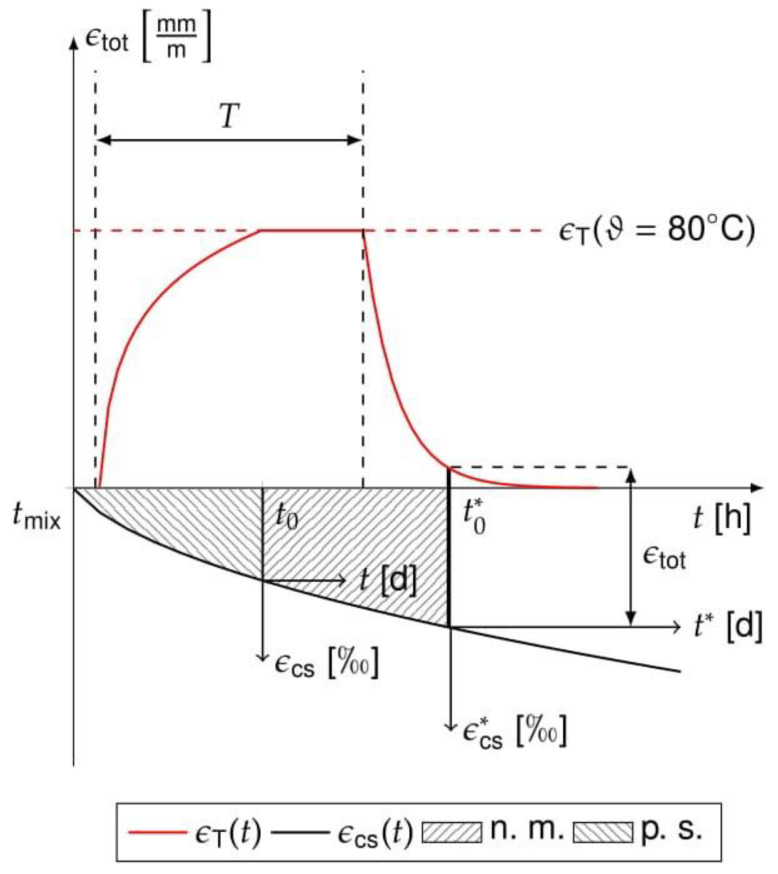
Temperature *ε*_T_ and shrinkage strains *ε*_cs_ during heat treatment.

The measured strains are superimposed contributions from shrinkage and temperature due to heat treatment (Figure 4). Plastic shrinkage (p. s.) starts when mixing the concrete at time *t*_mix_ and ends when hardened at *t*_0_. From *t*_0_ onwards, the volume reduction causes basic shrinkage or drying shrinkage ε_cs_. Both are caused by chemical or physical processes in the hardened pore structure of the concrete. The determination of *t*_0_, e.g., by the Vikat method [45], is not possible during heat treatment. The shrinkage strains after heating ε_cs_^*^ are determined from the time *t*_0_^*^ when the measuring marks are attached. Thereby, the plastic shrinkage as well as shares of autogenous and drying shrinkage are not measurable (n. m.). 

**Figure 5:**
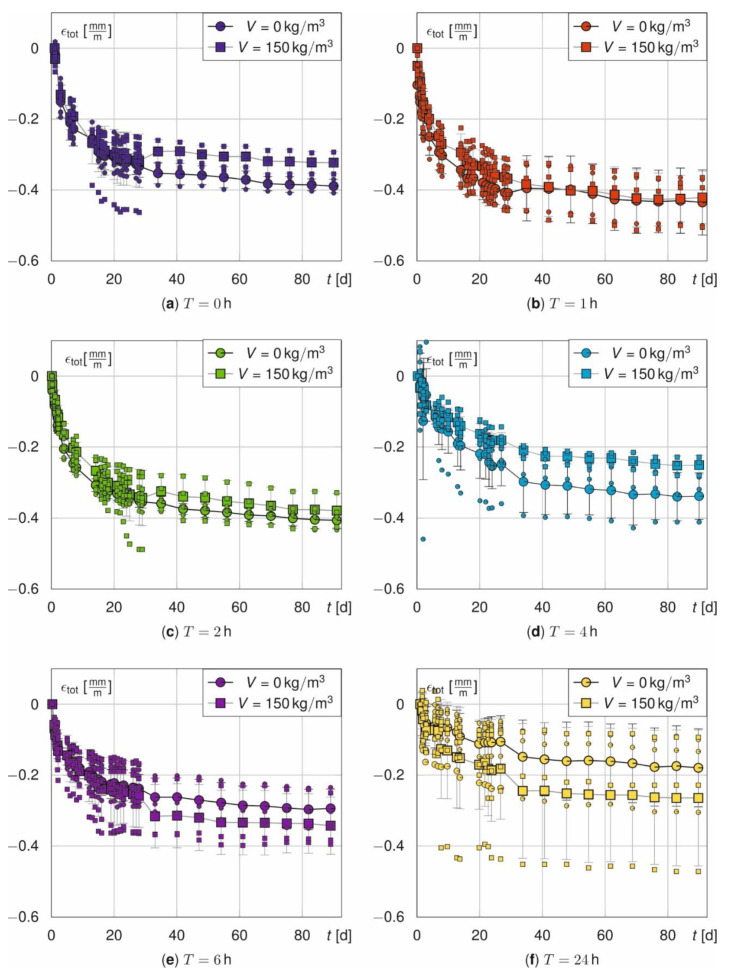
Total strain *ε*_tot_ of the prisms as a function of fibre amount *V* for T = 0 h (**a**), T = 1 h (**b**), T = 2 h (**c**), T = 4 h (**d**), T = 6 h (**e**) and T = 24 h (**f**).

**Figure 6:**
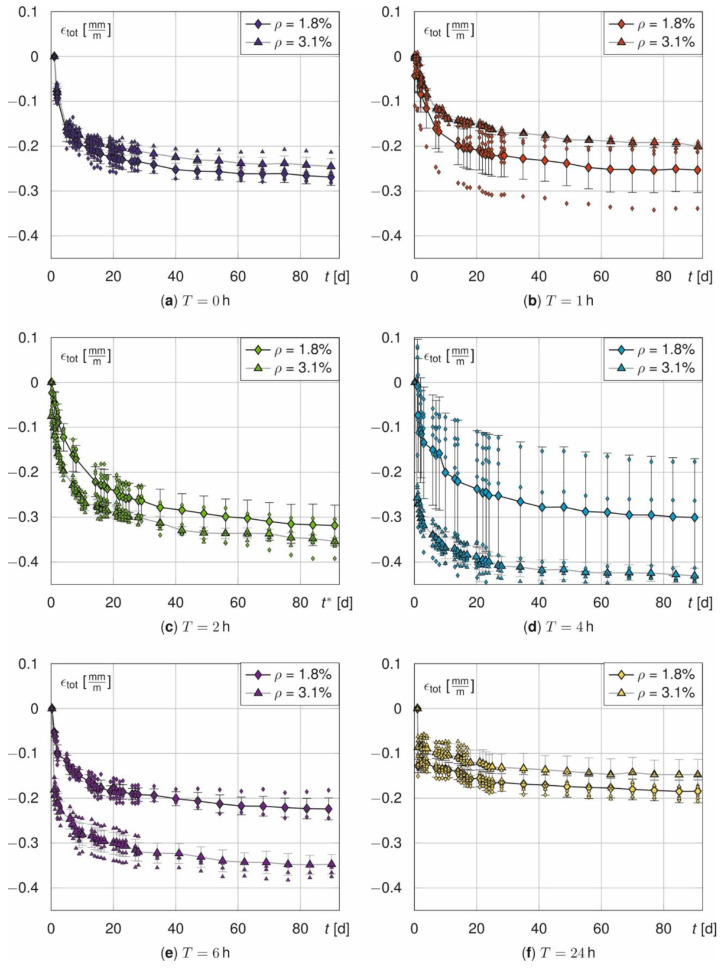
Total strain *ε*_tot_ of the beams as a function of rebar ratio *ρ* for T = 0 h (**a**), T = 1 h (**b**), T = 2 h (**c**), T = 4 h (**d**), T = 6 h (**e**) and T = 24 h (**f**).

Figure 5 and Figure 6 illustrate the measured total strains ε_tot_ over time. They are shown for both, prisms (Figure 5) and beams (Figure 6), as a function of the treatment time *T* with respect to fibre amount *V* and for beams also with respect to the rebar ratio *ρ*. In general, *ε*_tot_ increases strongly in the first days and seems to converge for both, prisms and beams, within 90 days. Additionally, the corresponding means and standard deviations are plotted as error-bars. The measured values of all samples scatter significantly due to the different initial temperature strains. The average standard deviations σ of the prisms are σ = [0.046, 0.049, 0.045, 0.024, 0.073, 0.131] σ = [0.032, 0.059, 0.019, 0.069, 0.039, 0.077] mm/m and mm/m for *T* = [0, 1, 2, 4, 6, 24] with and without fibres. *σ* unsystematically scatters. However, it tends to increase with *T*. Furthermore, σ is not constant over the time and increases abruptly for t^*^ > 28 d (see Figure 5a,e,f).

In general, the unsystematic scatter can be explained by superposed measurement inaccuracies from material properties and slightly changing storage conditions [46].

The discontinuity at t^*^ = 28 d in Figure 5 for the prisms arises from a jump in the number of samples. After 28 days, three out of six samples are used for the strength tests so that only three samples remain for the strain campaign (cf. Table 2). 

The standard deviations for the beams are *σ* = [0.019, 0.044, 0.033, 0.123, 0.015, 0.018] mm/m (*ρ* = 1.8%) and *σ* = [0.013; 0.005; 0.013; 0.021; 0.023, 0.028] mm/m (*ρ* = 3.1%) for *T* = [0; 1; 2; 4; 6; 24]. Only the beams with *ρ* = 3.1% show a systematic influence on *σ* that increases with increasing annealing time. The standard deviation of the beams decreases with an increasing rebar ratio and it is lower compared to the prisms with fibres. As the portion of (almost) deterministic materials, namely the reinforcement, increases, the residual portion of scattering material properties of the concrete decreases and, consequently, also the overall standard deviation does.

Consideration of the initial temperature strain *ε*_T_.

To determine the residual shrinkage strain after heat treatment *ε*_cs_^*^, the temperature strain *ε*_T_ according to Equation (3) is subtracted from the total strain *ε*_tot_.

(3) $$\varepsilon_{\mathrm{cs}}^{*}\left(\vartheta ,t\right)=\epsilon_{\mathrm{tot}}\left(\vartheta ,t\right)-\epsilon_{T}\left(\vartheta_{\mathrm{tg}},t_{0}^{*}\right)$$

The mean temperature of the samples *ϑ* is approximated using the Finite Element Method (FEM) [47]. The discretisation of the prisms in the program system ANSYS (2020 R2) [48] is done by eight nodes volume elements with trilinear shape functions. The reinforced bars are discretised according to [49].

As boundary conditions, the temperature ϑ in the specimen at time *t* = 0 h is set constant to the initial temperature ϑ_0_, according to Equation (4).

(4) $$\vartheta \left(h,t=0\right)=\vartheta_{0}$$

The heat transfer to the environment is simplified as free convection according to Equation (5) with the specimen temperature *ϑ* at the edge of the component geometry.

(5) $$-\lambda \frac{\partial \vartheta }{\partial h}=-\alpha \left[\vartheta_{\infty }-\vartheta \left(h=B/2,t\right)\right]$$

**Figure 7:**
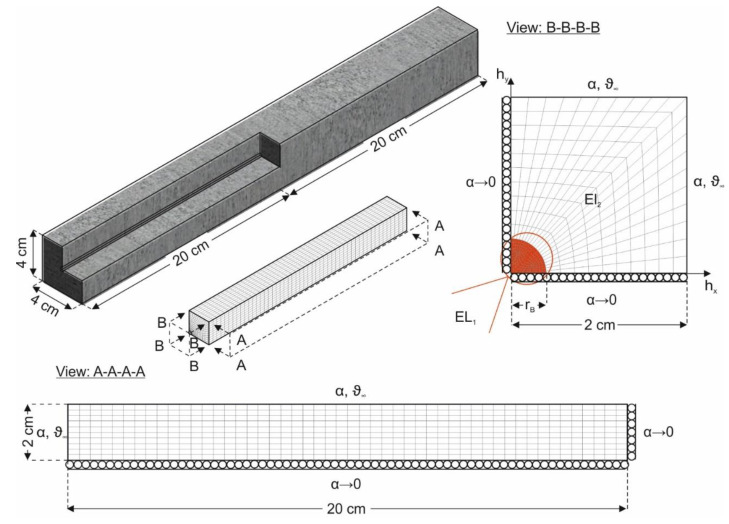
Discretisation of the beams.

The distance of the symmetry axis to the component edge corresponds to half the component width *B*. Figure 7 shows the numerical model for the thermal analysis of the beams. For this purpose, beam quarters exploiting the symmetry boundary conditions in the central axes with α → 0 W/(m² K) (insulated boundary) are used. The sections A-A-A-A and B-B-B-B represent the ambient temperature ϑ_∞_ and the heat transfer coefficient α as boundary conditions for the beam surfaces. Furthermore, section B-B-B-B shows the reinforcement elements (EL_1_) incorporated in the concrete, which are connected to the concrete elements (EL_2_) via the coupling conditions according to Equations (6) and (7), so that *ϑ* and the heat flux $\dot{q_{\lambda }}$ at the element transition at time *t* are the same. An element transition exists when the distance in the plane h_x_-h_y_ of the element position ***h*** to the axis origin corresponds to the radius of the rebar *r*_B_.

(6) $$\vartheta_{\mathrm{EL}1}\left(\left|h\right|=r_{B},t\right)=\vartheta_{\mathrm{EL}2}\left(\left|h\right|=r_{B},t\right)$$

(7) $${\dot{q}}_{\lambda ,\mathrm{EL}1}\left(\left|h\right|=r_{B},t\right)={\dot{q}}_{\lambda ,\mathrm{EL}2}\left(\left|h\right|=r_{B},t\right)$$

**Figure 8:**
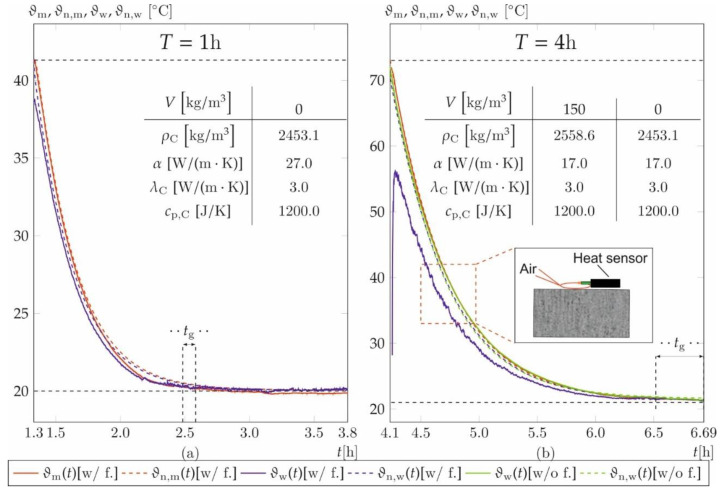
Comparison of measured core $\vartheta_{m}$ and surface temperatures $\vartheta_{w}$ along with the numerical expectations of core $\vartheta_{n,m}$ and surface temperatures $\vartheta_{n,w}$ of prisms for *T* = 1 h (**a**) and *T* = 4 h (**b**), respectively.

The numerical model [49] is validated by means of measured core *ϑ*_m_ and surface temperatures *ϑ*_w_ considered as Dirichlet boundary conditions. Figure 8 shows the strongly nonlinear temperature drops after stripping from initially 40 °C (a) and 70 °C (b) to room temperature (20 °C). Besides the empirical measurement, results of prisms with fibres (w/f.) and without fibres (w/o f.) are plotted along with the numerical results of the core *ϑ*_n,m_ and surface temperature *ϑ*_w,m_ exemplary for *T* equals 1 and 4 h. As mentioned, the prism without fibres for *T* = 1 h broke during stripping due to an insufficient early strength. Hence, no temperature data are available.

To account for the numerical deviations of the temperatures, the heat transfer coefficient *α* is adjusted so that the relative deviation of the numerical core temperature *ϑ*_n,m_ and *ϑ*_m_ in the period *t*_g_ is less than 1 °C. The period *t*_g_ implies all start times *t*_0_^*^ of the prisms with and without fibres as well as the beams depending on the rebar ratio *r* and is different for each temperature duration *T*. It defines the time required to glue the markers for deformation measurements and lasts up to 20 min. *ε*_T_ is calculated with a coefficient of thermal expansion *α*_C_ = 12 × 10^−6^ m/K [37].

The heat capacity and thermal conductivity for the concrete elements EL_2_ is set to *c*_p,C_ = 1200 J/K and *λ*_C_ = 3 W/(mK) according to [37], which correspond approximately to the thermal properties of already hardened concrete. The density of the prisms *ρ*_C_ is measured and listed with the thermal properties in Figure 8. The thermal properties of the reinforcement elements (EL_1_) are set to *λ*_S_ = 50 W/(m K), *ρ*_S_ = 7800 kg/m³ und *c*_p,S_ = 450 J/(kg K) for reinforced steel acc. to [47].

The relative deviations between numerical approximation of *ϑ*_n,m_ and measured core temperatures *ϑ*_m_ are [-, 4.8, 0.9, 0.5, 0.7]% without fibres and [1.2, 1.6, 4.8, 0.1, 2.3]% with fibres for *T* = [1, 2, 4, 6, 24] h during time period *t*_g_. The relative deviations between the surface temperature *ϑ*_n,w_ and *ϑ*_w_ from numerical simulation are [-, 3.1, 1.6, 2.0, 5.5] without fibres and [0.5, 1.7, 4.5, 1.8, 1.5]% with fibres.

The temperature deviations remain below 1 °C for all temperature durations *T*, except for *T* = 24 h (without fibres) where the temperature deviation yields 1.2 °C. 

It must be noted that the numerical solutions of temperature durations *T* ≠ 1 h deviate from the measured values at *t* < *t*_g_ (c.f. Figure 8b). This is attributed to measurement errors caused by the small air pads between concrete surface and heat sensor (Figure 8b, detail). Already small differences in the height of the sensors lead to large temperature deviations, since the temperature of the resulting air layer is then measured instead of the surface temperature.

**Figure 9:**
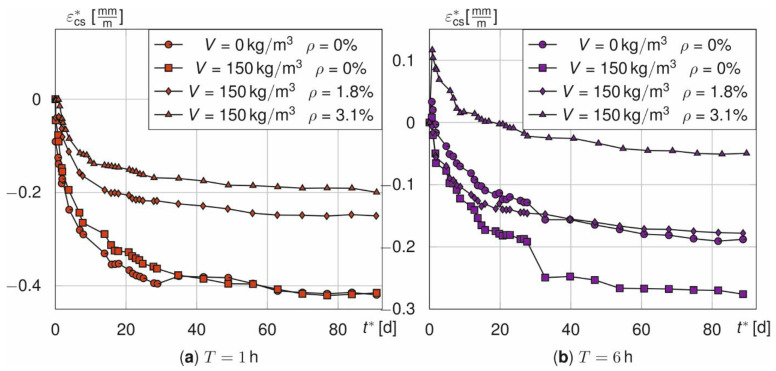
Shrinkage strain *ε*_cs_^*^ of prisms and beams for *T* = 1 h (**a**) and *T* = 6 h (**b**).

However, the more relevant behaviour after cooling is not affected. Compared to the time-dependent course of ε_cs_^*^ according to Equation (3) for treatment times of 1 h (Figure 9a) and 6 h (Figure 9b) the shrinkage curves show two physical discrepancies. On the one hand, the halved number of samples causes discontinuities at t^*^ = 28 d. This is illustrated by the abrupt decrease or increase of *ε*_cs_^*^. On the other hand, a supposed swelling occurs for prisms without fibres at *T* = 1 h that is not physically justifiable. Furthermore, the influence of *ε*_T_, especially for the beams, leads to positive values of ε_cs_^*^ at time t^*^ = 0 d. The reason for this is that the numerical model overestimates the average temperature in the specimen. This holds especially true for the beams with a reinforcement ratio of 3.1% (Figure 9b). 

Shrinkage curve adjustments

Physically unreasonable curves are adjusted in two aspects: First, the values are harmonised after 28 d; second, the origin is shifted. 

**Figure 10:**
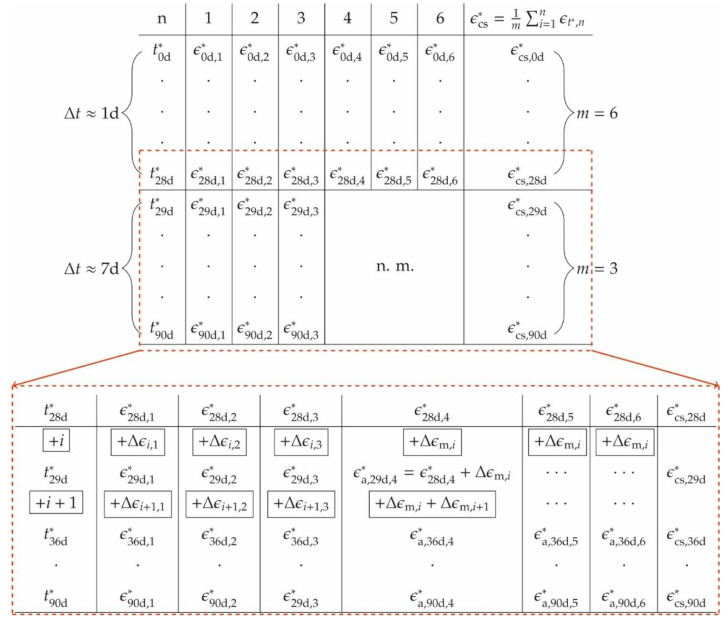
Summary of the shrinkage measurement of *ε*_cs_^*^ and the adjustment for t^*^ > 28 d.

To smoothen the discontinuities in the strain curves at t^*^ = 28 d caused by the halved number of specimens, averaging is applied. The approach is schematically shown in Figure 10. In the upper part of Figure 10, the variables of the shrinkage strains of the first 28 days are plotted. For each individual prism *n* the shrinkage strains ε^*^_t*,n_ are determined from an interval Δ*t* of approximately 1 d. This is done by the mean from *m* = 6 samples. 

The lower part in Figure 10 shows the variables of the measured values after 28 days. Specimens *n* = 4–6 have been extracted for strength evaluations and thus only samples *n* = 1–3 remain. Moreover, the time interval Δ*t* of measurements is increased to 7 days. Missing shrinkage strains (*n* = 4–6) are interpolated, assuming constant strain increases Δ*ε* within each time increment *i* = Δ*t*. The calculative shrinkage strains ε^*^_t*,n_ for *n* = 4–6 are estimated by adding average strains Δ*ε*_m,*i*_ of the samples *n* = 1–3 to the last measured strains *ε*_28d,n_^*^. For the time increments *i*+1, the sum of all Δ*ε*_m,*i*_ is taken. Thus, the averaged total strain is calculated from three measured strains (*n* = 1–3) and three interpolated ones (*n* = 4–6). 

**Figure 11:**
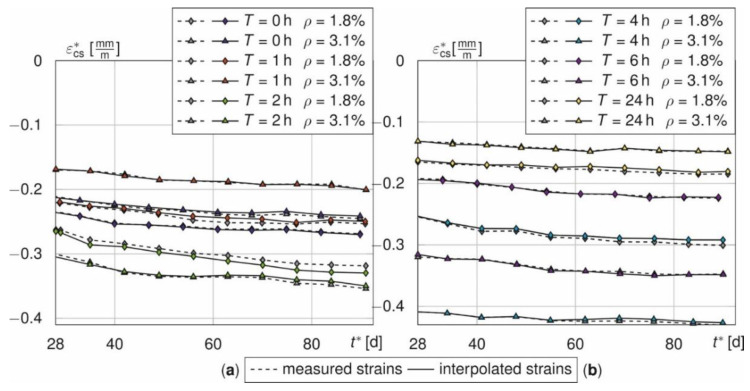
Comparison of the interpolated (colours) and measured (grey, dashed) total strains of the beams for *T* = 0, 1, 2 h (**a**) and *T* = 4, 6, 24 h (**b**).

The adjustment scheme is controlled using the total strains of the beams, since the shrinkage strains were measured up to 90 days for all 6 samples. Therefore, the strains *ε*_t*,4–6_^*^ for *t* > 28 d are set to zero and then interpolated. Figure 11 represents the time course of the shrinkage strain of the beams with and without interpolation of the measured values *n* = 4–6. Shown are the interpolated (colours) and the measured shrinkage strains (grey, dashed) for *T* = [0, 1, 2] h (a) and *T* = [4, 6, 24] h (b). Both curves do almost coincide. 

The interpolated strains show maximum relative deviations of [0.7, −3.0, 3.4, −3.0, 1.2; −3.2]% (*ρ* = 1.8%) and [−1.8, 1.5, −1.6, −1.1, −1.3, 1.9]% (*ρ* = 3.1%) for *T* = [0, 1, 2, 4, 6, 24] h. The results indicate that the shrinkage behaviour of the bars can be approximated even with a reduced number of specimens. The same applies to the prisms. 

Thus, the fitted values represent the true shrinkage behaviour more likely than the discontinuous curves.

A shift of the origin is applied to all shrinkage curves that do not start from zero strain at time t^*^ = 0 d after calculated thermal shares are removed. This happens for beams with *ρ* = 3.1% and *T* = 4 and 6 h. 

**Figure 12:**
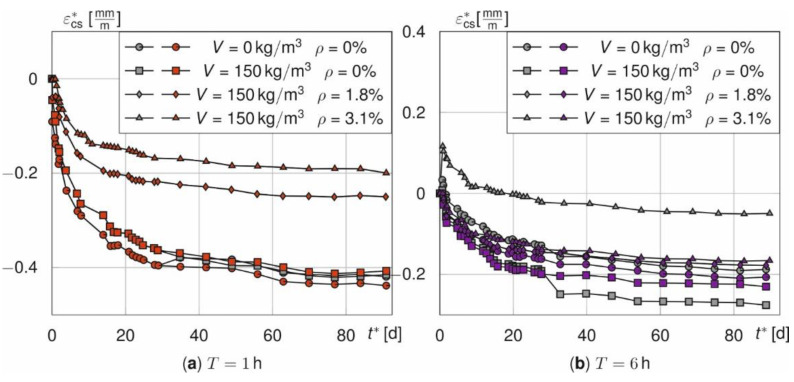
Comparison of the adjusted and shifted (red, purple) as well as the measured shrinkage curves (grey) of prisms and beams for *T* = 1 h (**a**) and *T* = 6 h (**b**).

Figure 12 shows the strain courses *ε*_cs_^*^ for the different fibre and rebar amounts over the next 90 days when the two adjustments are made (in red for *T* =1 (a) and in purple for *T* = 6 h (b)) relative to the originally determined values in grey. Discontinuities at t^*^ = 28 d have disappeared and now all curves start from zero. Thus, these two adjustments are performed for all following analyses.

Evaluation of shrinkage strains ε_cs_^*^

**Figure 13:**
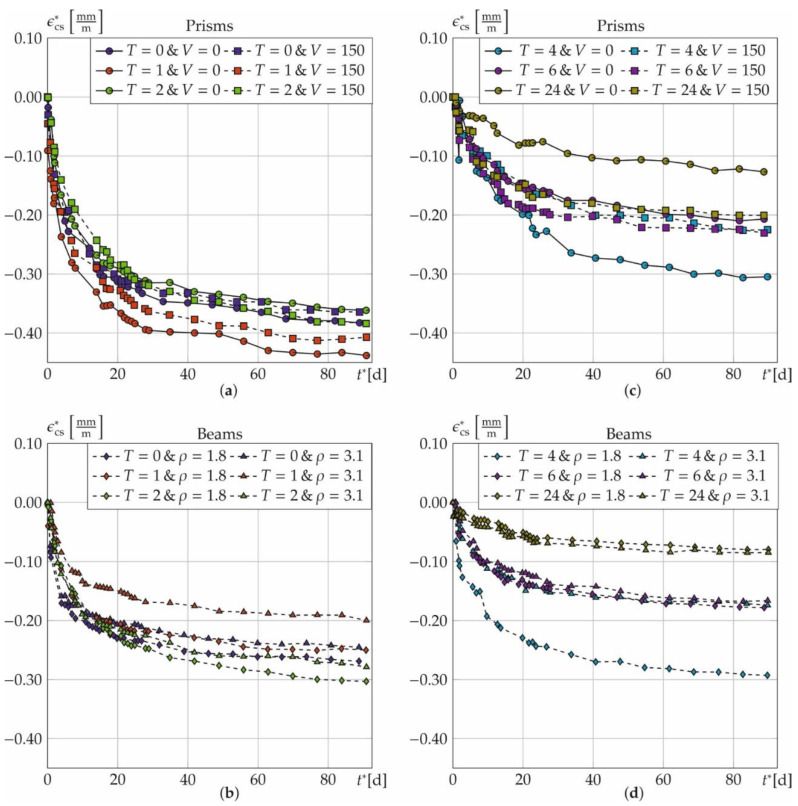
Results of the shrinkage strain *ε*_cs_^*^ as a function of the fibre amount *V* [kg/m³] for the prisms for *T* = 0, 1, 2 (**a**) and *T* = 4, 6, 24 (**b**) or rebar ratio *ρ* [%] for the beams for *T* = 0, 1, 2 (**c**) and *T* = 4, 6, 24 (**d**).

Figure 13 shows the asymptotic courses of the shrinkage strains for prisms (top) and beams (bottom) with time. The single curves represent the average values of the residual strain *ε*_cs_^*^ at concrete ages t^*^. They are plotted for prisms at *T* = [0, 1, 2] h (left) and *T* = [4, 6, 24] h (right) with and without steel fibres. The same is done for the beams (bottom) while the geometric rebar ratio is kept constant at 1.8%. For all specimens—regardless of the treatment time, the fibre amount or the rebar ratio—shrinkage rises after heat treatment. However, it nearly converges after about 60 days. This is attributed to the latent-hydraulic blast furnace slag contained in the binder, giving rise to a slower hydration of the concrete [50]. *ε*_cs_^*^ results to 0.383 mm/m after 90 days for the reference prisms. Fibre addition slightly lowers the strain to 0.364 mm/m due to stiffening through a higher Young’s modulus of FRC [13]. *ε*_cs_^*^ generally decreases from heating. Reductions amount to [5.7, 20.4, 45.9, 66.8]% for *T* = [2, 4, 6, 24] h in case of no fibres due to accelerated hydration. Remember, that the strength tests in Section 3.3 revealed that hydration is almost completed after *T* = 24 h. This is also supported by a very low shrinkage rate of the prisms without fibres (top, right). Due to the high degree of hydration, the residual shrinkage strains most likely result from drying.

Heat treatment also reduces shrinkage of specimens with fibres (prisms), but to a lower extent. Reductions amounts to [38.3, 36.7, 44.9]% for *T* = [4, 6, 24] h. A treatment time of *T* = 2 h has a negligible effect on the strains. It induces an increase of 5.3%. The prisms with treatment time equal to *T* = 1 h show higher shrinkage of 14.4% (without fibres) and 11.7% (with fibres) compared to the reference samples. These samples were still very moist after heating. Hence, it seems reasonable that the additional shrinkage results from drying. Comparing prisms with and without fibres and equal treatment times the shrinkage strains decrease by [7.7, 35.5]% for *T* = [1, 4] h and increase by [5.9, 10.3, 36.7]% for *T* = [2, 6, 24] h showing an unsystematic combined impact of fibres and heat treatment. 

On the one hand, steel fibres increase the stiffness and reduce the overall shrinkage strain. Moreover, they reduce the activation energy of the concrete [34], so that the cement hydrates quicker [51]. This lowers autogenous shrinkage, since larger portions of the strain are anticipated already during heat treatment. 

On the other hand, steel fibres can enhance drying shrinkage as pores are connected and water permeability increases [17]. The increased porosity in the contact zone of cement matrix and steel fibres [16] can even enhance this effect.

Beams without heat treatment exhibit significantly lower shrinkage strains of 0.27 mm/m (*ρ* = 1.8%), and 0.24 mm/m (*ρ* = 3.1%) compared to the fibre-reinforced prisms. The longitudinal reinforcement acts as a “shrinkage brake” proportional to the additional stiffness induced by rebar [52]. Heat treatment reduces shrinkage strains up to a factor of about three, namely by [7.0, 33.8, 70.6]% for *T* = [1, 6, 24] h and *ρ* = 1.8%. For *ρ* = 3.1% this effect is slightly reduced and amounts to [18.5, 29.1, 32.2, 65.5]% for *T* = [1, 4, 6, 24] h. Shrinkage of beams with higher rebar ratios (*ρ* = 3.1%) is 6.7 to 40.6% smaller compared to those with *ρ* = 1.8%. This holds true for *T* = [0, 1, 2, 4, 6] h. However, even slightly inverse trends appear (*T* = [2, 4] h and *ρ* = 1.8% as well as *T* = 2 h and *ρ* = 3.1%) what underlines the inherent uncertainty caused by all measuring techniques and separating temperature strains calculative.

### 3.3. Flexural and Compressive Strength

**Figure 14:**
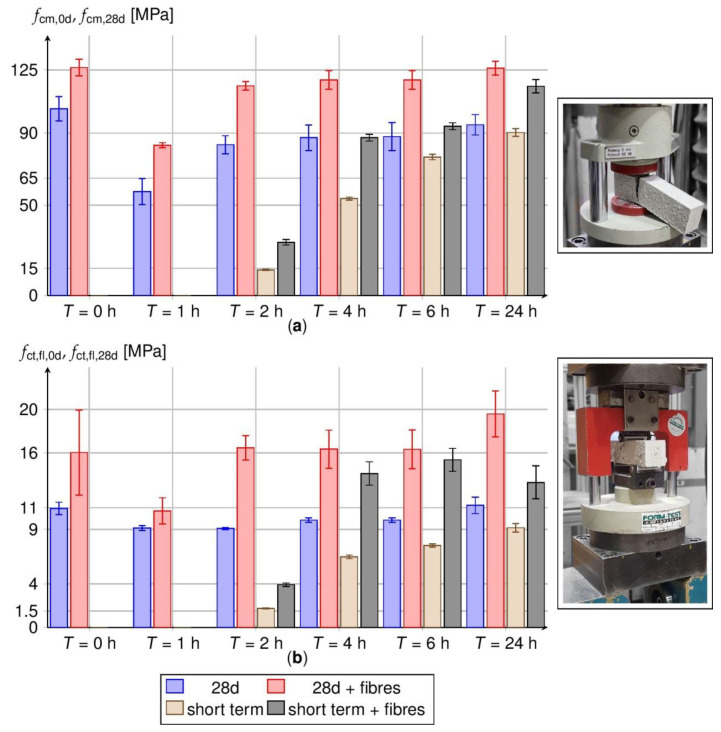
Compressive- (**a**) and flexural strengths (**b**) of the prisms at *t* = 28 d (reference, red and blue columns) and directly after the heat treatment (brown and grey columns).

Flexural and compressive strength tests are performed on the prisms using standard testing techniques (cf. Figure 14, right). The results are plotted in bar diagrams (cf. Figure 14, left) as functions of *T* and *V* and compared to reference samples with or without steel fibres. Short-term tests are performed directly after heating (brown and grey bars). Reference tests are conducted after 28 days and the plotted initial heat treatment times (red and blue bars). Compressive strengths are presented by means *f*_cm_ and scattering ranges in the top diagram, flexural strength values *f*_ct,fl_ according to the same scheme but in the bottom diagram. 

The reference specimen without heat treatment exhibits a mean compressive strength *f*_cm,28d_ of 103.5 MPa. Additional steel fibres increase the strength by 22.1% to 126.3 MPa. The increase is mostly attributed to prevented lateral expansion by the fibres [53]. Generally, heat treatment reduces strength. *f*_cm,28d_ decreases by [44.3, 19.3, 15.5, 14.9, 8.6]% for *T* = [1, 2, 4, 6, 24] h with no fibres. Obviously, the loss turns out less pronounced with increasing treatment time *T*. Especially, the steep rates *R*_a_ > 20 K/h in the first two hours of heating induce internal stresses in the cement matrix that provoke structural damage. With increasing *T*, temperature gradients and thus the stresses reduce. This promotes also the formation of further hydration products that bridge microcracks [4].

Fibres help to reduce damage induced by heating. They bridge microcracks [31]. The samples also exhibit lower compressive strengths, but less distinct. The decrease yields [34.1, 8.0, 5.4, 5.4]% for *T* = [1, 2, 4, 6] h. For *T* = 24 h the compressive strength is almost the same in the reference sample. 

The standard deviation of the compressive strength lies between 5.0 and 7.8 MPa (no fibres)—so slightly above the expectation for laboratory concrete [54]—and decreases to 1.5 to 5.2 MPa, if fibres are added. The scatter generally seems independent of the heat treatment time *T*. As for the strength, fibres are proven to reduce scatter and homogenise the inner strength state by bridging cracks [31].

Reasonable compressive strength develops after about 2 h of heating (Figure 14, grey and brown columns). It amounts to *f*_cm,0d_ = [14.2, 53.7, 76.8, 90.3] MPa for *T* = [2, 4, 6, 24] h in case of pure HPC and [29.5, 87.5, 93.8, 116.0] MPa if fibres are added. One-day long heat treatment (24 h) almost completes hydration and hardening. Only 4.7 and 8.6%, respectively, of the long-term strength after 28 days is missing after 24 h. 

The reference value of the flexural strength *f*_ct,fl,28d_ yields 10.9 MPa after 28 d. It holds true for pure HPC without heating. Heat treatment reduces this strength by [16.4, 16.8, 9.7, 9.7]% to [9.1, 9.1, 9.9, 9.9] MPa for *T* = [1, 2, 4, 6] h. On the contrary, 24 h heating gives rise to keep the strength almost constant (+2.7%). 

Steel fibres significantly increase the flexural strength by activating the pull-out resistance [55]. This holds true for all heating regimes and time stages investigated. The specific HPC with particle sizes down to nano-scale provides the necessary adhesion to the smooth steel surfaces [37]. The reference sample achieves *f*_ct,fl,28d_ = 16.0 MPa, what exceeds the value of the pure HPC by 46.9%. 

Heat treatment just reduces the flexural strength on the short run for *T* = 1 h (10.7 MPa). In any other situation (*T* > 1 h), strength is enhanced up to 22.1% for *T* = 24 h. 

The standard deviation of the flexural strength with fibres is [3.9, 1.2, 1.1, 1.7, 1.8, 2.1] MPa for *T* = [0, 1, 2, 4, 6, 24] h and lies above the strengths for prisms without fibres. This is mainly due to the scattered distribution and orientation of fibres [32,33,56] that is subjected to wall effects from the pouring process [57]. As the ratio of the cross-sectional width (40 mm) to the fibre length (13 mm) is small, fibres clearly orientate along the longitudinal prism axis and do not distribute isotropic. 

In general, an addition of steel fibres is recommended for short-term heat treatments without consideration of prescribed heating rates and pre-storage times. That way a large share of the strength losses is compensated. Moreover, stripping of formwork might start early after *T* > 2 h; then sufficient short-term strength has already developed. The standard deviation of the flexural strength slightly increases by an addition of steel fibres.

## 4. Conclusions

The influence of rapid heat treatment of 80 °C on the shrinkage behaviour and strength of HPC is investigated. The experimental investigations cover different treatment times between 0–24 h as well as the steel fibre amounts and rebar ratios. The main findings are as follows:

- With increasing treatment time *T*, the shrinkage strains decrease. For *T* = 24 h it is reduced to 66.8% (no fibres) or 44.9% (fibres) compared to the reference samples without heat treatment (0.383 mm/m without steel fibres and 0.364 mm/m with steel fibres for *T* = 0 h).
- Short treatment times of *T* = 1 to 2 h have no beneficial or even slightly negative effects on shrinkage strains. Heat treatment durations greater than 2 h must be selected to improve the shrinkage behaviour.
- For *T* = 4 and 6 h, the residual shrinkage strains can be reduced up to 0.176 mm/m for prisms and 0.09 mm/m for beams what corresponds to 45.9% and 33.8% compared to the reference samples without heat treatment. However, the investigations show high scatter for different treatment times and specimen types.
- Rebar serves as a “shrinkage brake”. Reinforcing bars reduce shrinkage proportional to its longitudinal stiffness.
- The compressive strength of the prisms decreases up to 44% for *T* = 1 h. To prevent losses of compressive strength due to structural damage, tempering of *T* ≥ 2 h is recommended.
- An addition of steel fibres significantly increases the compressive strength as well as the flexural strength, as fibres prevent structural damage in the matrix induced by internal stresses.
- For practical application, the use of steel fibres and reinforcement is recommended to improve the shrinkage behaviour.
- For practical applications it is recommended to use tempering at around 80 °C with durations greater than 2 h. Durations of 2 to about 6 h are most effective. Durations exceeding 24 h are not recommended, as the beneficial effect on shrinkage more and more decreases over time and then reaches a plateau. Micro steel fibres should be added to preserve the bearing abilities of the HPC that otherwise noticeably decreases by temperature induced constraints.

## Figures and Tables

**Table 1:** Concrete mix of high-performance concrete.

Component	Type	Mass [kg/m³]
River sand	0/2	426.0
Crushed stone basalt	1/3	882.0
Binder	Nanodur^®^ Compound 5941	1042.0
Water	-	159.8
Superplasticizer	Master Glenium ACE 430	12.3
Shrinkage reducer	Eclipse Floor	8.0
Hardening accelerator	Master X-Seed 100	12.3
Steel fibres	*d*/*l* = 0.19/13 [mm]	0/150.0

**Table 2:** Summary of performed tests and ratio of the tested and the total number of samples, denoted by tested/total number of prisms/beams.

Experimental Set-Ups	Concrete Age *t*	Test Specimens			
		Prisms *V* = 0 kg/m³	Prisms V = 150 kg/m³	Beams *ρ* = 1.8%	Beams *ρ* = 3.1%
Temperature measurement	≤1 d	6/36	6/36	-	-
Shrinkage	≤28 d	36/36	36/36	36/36	36/36
	>28 d	18/36	18/36	36/36	36/36
Compressive strength	≈0 h	12/12	12/12	-	-
	28 d	18/36	18/36	-	-
Flexural strength	≈0 h	12/12	12/12	-	-
	28 d	18/36	18/36	-	-

## Data Availability

Data is contained within the article.
